# Advances of neuroimaging findings in medication overuse headache

**DOI:** 10.3389/fnins.2025.1715175

**Published:** 2025-12-10

**Authors:** Xiaoni Wang, Dan Zhang, Yi Yang, Jiaxin Zheng, Mengze Jiang, Jin Wang

**Affiliations:** Headache Center, Department of Neurology, Sir Run Run Shaw Hospital, School of Medicine, Zhejiang University, Hangzhou, China

**Keywords:** medication overuse headache, neuroimaging, pain matrix, mesocorticolimbic reward circuitry, addiction

## Abstract

Medication overuse headache (MOH) is a chronic secondary headache disorder that develops from excessive analgesic use. The prevalence of MOH is estimated 1–2% in the general population, reaching up to 50% among individuals with chronic headache. Although the pathophysiological mechanisms underlying MOH remain incompletely understood, neuroimaging studies have provided valuable insights into its developments. This review synthesizes current evidence demonstrating that MOH is associated with structural and functional alterations in two key neural systems: (1) the pain matrix involved in nociceptive processing, (2) the mesocorticolimbic reward circuitry implicated in addictive behaviors. Importantly, certain structural and functional changes show partial reversibility following medication withdrawal. Nevertheless, longitudinal studies with larger sample sizes are necessary to establish causal relationships between these neurobiological changes and the development/maintenance of MOH, which remains essential for developing targeted interventions.

## 1 Introduction

Medication overuse headache (MOH) is a chronic secondary headache disorder that develops from excessive use of acute headache medications. MOH is characterized by headaches occurring at least 15 days per month in patients with a pre-existing headache disorder and regular intake of analgesics for more than 10 or 15 days per month for >3 months, depending on the class of medication being overused. The prevalence of MOH is estimated at 1–2% in the general population, and up to 50% among patients with chronic headache (Stovner et al., 2007). Despite its clear clinical definition, the underlying neurobiological mechanisms remain poorly understood, limiting the development of targeted and effective treatments.

Emerging evidence suggests that MOH involves complex interactions between neural adaptation, genetic susceptibility, and environmental factors (Ashina et al., 2023). Chronic medication exposure is hypothesized to induce central sensitization and alterations in pain-processing pathways (Diener et al., 2019; Sebastianelli et al., 2023). Psychological studies indicate that MOH and drug addiction share overlapping psychiatric comorbidities, personality traits, and maladaptive reward circuitry (Takahashi et al., 2021).

Recent advances in neuroimaging techniques have enhanced our understanding of the neuromechanisms underlying MOH. Multiple imaging modalities, including structural magnetic resonance imaging (MRI) for assessing gray matter morphology and white matter integrity via diffusion tensor imaging (DTI), functional MRI (fMRI) for mapping dynamic neural activity and connectivity, and positron emission tomography (PET) for evaluating neurotransmitter system dysregulation and brain metabolism, have been systematically employed.

**Figure 1:**
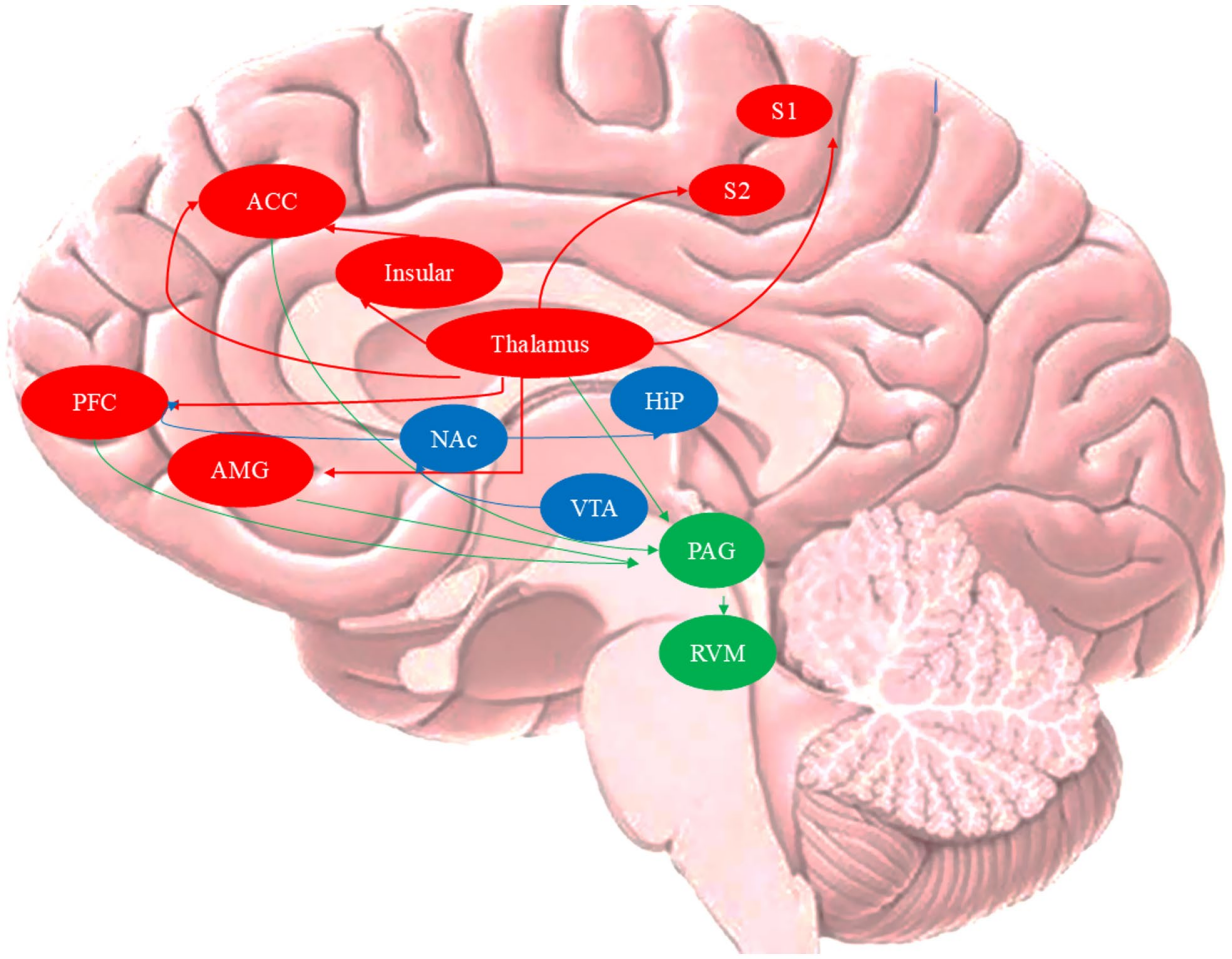
Anatomy of the pain neuromatrix and mesocorticolimbic dopaminergic circuit. VTA, ventral tegmental area; HiP, hippocampus; NAc, nucleus accumbens; S1, primary somatosensory cortex; S2, secondary somatosensory cortex; ACC, anterior cingulate cor tex; PFC, prefrontal cortex; AMG, amygdala; PAG, periaqueductal gray; RVM: rostral ventromedial medulla.

This review aims to synthesize and critically evaluate the growing body of neuroimaging literature on MOH. We systematically examine studies comparing MOH patients with episodic migraineurs (EM), chtonic migraineurs (CM) and normal controls (NC), particularly regarding pain-processing related brain regions (“pain matrix”) and the mesocorticolimbic dopamine circuitry (reward-system) (Figure 1). Furthermore, we explore brain mechanisms of recovery following medication withdrawal. By integrating multimodal evidence from neuroimaging studies, this work seeks to clarify the neural substrates of MOH (Table 1).

**Table 1:** Summary of neuroimaging findings in patients with medication overuse headache.

References	Subject cohorts	Analysis	Medication class	Comparison group	Main results
Riederer et al. (2012)	MOH (*n* = 29) NC (*n* = 29)	sMRI: VBM	Simple analgesics Triptans Combination analgesics Opioids	MOH vs. NC	Increased GMV in periaqueductal grey, bilaterally thalamus, and ventral striatum Decreased GMV in frontal regions including orbitofrontal cortex, anterior cingulate cortex, left and right insula, and precuneus
Chen et al. (2017c)	MOH (*n* = 27) NC (*n* = 27)	sMRI: VBM	NR	MOH vs. NC	Increased GMV in the whole thalamus and each thalamic subnuclei, negatively related with depression
Ferraro et al. (2012a)	MOH (*n* = 9, immediately and 6 months after withdrawal) NC (*n* = 9)	Task-fMRI: Noxious heat stimulation	NSAIDs (*n* = 3) NSAIDs + Triptans (*n* = 4) NSAIDS + Triptans + Caffeine (*n* = 2)	MOH VS NC Before vs. after withdrawal	Hypoactivity of the primary somatosensory cortex, inferior parietal lobule, and supramarginal gyrus at the beginning after withdrawal Recovered to almost normal activity 6 months after withdrawal
Grazzi et al. (2010)	MOH (*n* = 9, before and 6 months after withdrawal) NC (*n* = 11)	Task-fMRI: Tonic mechanical pressure stimulation	NSAIDs Combination analgesics	MOH vs. NC Before vs. after withdrawal	Hypoactivity of the right supramarginal gyrus, the right inferior and superior parietal cortex before withdrawal Recovered to almost normal activity 6 months after withdrawal
Chen et al. (2017a)	MOH (*n* = 37) EM (*n* = 18) NC (*n* = 32)	Rs-fMRI	Simple analgesics (*n* = 34) Triptans (*n* = 1) Combination analgesics (*n* = 2)	MOH vs. NC MOH vs. EM	Decreased FC density in right parahippocampal gyrus, and increased FC density in left inferior parietal gyrus and right supramarginal gyrus; decreased FC in the right prefrontal cortex, frontopolar cortex Decreased FC density of right caudate and left insula and decreased fronto-temporal connectivity
Fumal et al. (2006)	MOH (*n* = 16, before and 3 weeks after withdrawal) NC (*n* = 68)	FDG-PET	Non-narcotic analgesics, chiefly paracetamol (*n* = 8) Combination analgesics (*n* = 6) Ergotamine-caffeine preparation (*n* = 2).	MOH vs. NC Before vs. after withdrawal	Hypometabolism in the bilateral thalamus, anterior cingulate gyrus, insula/ventral striatum and right inferior parietal lobule, hypermetabolism in the cerebellar vermis Persistent hypometabolism of orbitofrontal cortex after withdrawal
Niddam et al. (2020)	MOH (*n* = 16) CM (*n* = 16) NC (*n* = 16)	MRS	NR	MOH vs. CM	Higher glutamate and glutamine in the right mid cingulate cortex and myo-inositol in the left anterior cingulate cortex
Lai et al. (2012)	MOH (*n* = 30) CM (*n* = 23) EM (*n* = 19) NC (*n* = 16)	MRS	NR	MOH vs. CM	No significant differences
Riederer et al. (2017)	MOH (*n* = 29) NC (*n* = 29)	sMRI: SBM, cortical thickness, gyrification	Simple analgesics (*n* = 21) Triptans (*n* = 20) Combination analgesics (*n* = 3) Opioids (*n* = 3)	MOH vs. NC	Reduced cortical thickness in the left prefrontal cortex and higher local gyrification from the fusiform cortex to medial temporal regions, and in the right occipital pole. Higher gyrification in the right occipital pole predicted poor response after detoxification
Bai et al. (2023)	MOH (*n* = 33) CM (*n* = 22) EM (*n* = 26) NC (*n* = 26)	pCASL MRI: CBF, aCBV	NR	CM vs. NC MOH vs. CM	Increased CBF and aCBV values in the left amygdala and increased CBF values in the right amygdala No significant differences
Chen et al. (2017b)	MOH (*n* = 22) NC (*n* = 22)	sMRI: VBM	NR	MOH vs. NC	Increased volume of periaqueductal gray
Chen Z. Y. et al. (2018)	MOH (*n* = 36) NC (*n* = 32)	sMRI: VBM	NR	MOH vs. NC	Increased volume in the left ventrolateral periaqueductal gray, ventral tegmental area, bilateral substantia nigra, and trigeminal root entry zone
Chen et al. (2016)	MOH (*n* = 44) CM (*n* = 16) EM (*n* = 18) NC (*n* = 32)	Rs-fMRI	Simple analgesics (*n* = 3) Triptan (*n* = 1) Opioids (*n* = 1) Combination analgesics (*n* = 33) Multiple drug classes (*n* = 6)	CM vs. EM MOH vs. EM MOH vs. CM	Decreased FC and increased FC of neostriatum Decreased FC of neostriatum Increased FC of neostriatum
Liu M. et al. (2024)	MOH (*n* = 29) NC (*n* = 29)	PCASL MRI: CBF	NR	MOH vs. NC	Decreased perfusion in the left posterior, left tubular superior, right anterior–inferior, right tubular inferior, right tubular superior, right posterior subunit and the entire right hypothalamus
Chanraud et al. (2014)	MOH (*n* = 17 and 9) EM (*n* = 18 and 15) NC (*n* = 17 and 17)	sMRI: VBM Rs-fMRI	Triptan (*n* = 4) Simple Analgesic (*n* = 2) Opioid (*n* = 2) Combination analgesic (*n* = 1) Combination of medication (*n* = 8)	MOH vs. EM	Decreased FC between precuneus and regions of the default-mode network, increased FC between precuneus and hippocampal/ temporal areas No significant differences in morphological changes.
Niddam et al. (2023)	MOH (*n* = 26) CM (*n* = 27) NC (*n* = 26)	Task-based MRI: intertemporal choice task Rs-fMRI	Acetaminophen Caffeine NSAIDs Eergotamine	MOH vs. NC MOH vs. CM and NC	Weaker subjective value representations in the dorsomedial prefrontal cortex when accepting larger delayed rewards, and in ventral striatum and ventromedial prefrontal cortex when accepting the smaller immediate reward. Reduced resting-state functional connectivity among the valuation regions
Torta et al. (2016)	MOH (*n* = 15) Non-MOH (*n* = 15)	Rs-fMRI	Triptans + Analgesics (*n* = 2) Triptans (*n* = 7) Combination Analgesics (*n* = 3) Analgesics (*n* = 10) Analgesics + Combination (*n* = 1) Combination analgesics+ Opioids (*n* = 1) Triptans + FANS (*n* = 1) NSAIDs (*n* = 1)	MOH vs. non-MOH	Nucleus accumbens and dorsal rostral putamen functional connectivity could discriminate between MOH and non-MOH patients
Tantik Pak et al. (2021)	MOH (*n* = 25) CM (n = 33)	DTI	NR	MOH vs. CM	Significant difference in FA values of the left OFC between the two groups
Lai et al. (2016)	MOH (*n* = 33) CM (*n* = 33) NC (*n* = 33)	sMRI: VBM	NR	MOH vs. CM	Decreased GM in the orbitofrontal cortex and left middle occipital gyrus as well as increased GM in the left temporal pole/parahippocampus
Liu H. et al. (2024)	MOH (*n* = 17) NC (*n* = 16)	DAT-PET	Combination analgesics (*n* = 16) Triptan (*n* = 1)	MOH vs. NC	Lower Suvr levels in the medial rather than lateral orbitofrontal cortex
Riederer et al. (2013)	MOH (*n* = 22, 11) responders, 11-non-responders, before and 3 months after withdrawal	sMRI: VBM	Triptans (*n* = 6) Simple analgesics (*n* = 7) Triptans + Simple analgesics (*n* = 8) Triptans + combination analgesics (*n* = 1)	Before and after withdrawal Responders vs. non-responders	Decrease of previously increased gray matter in the midbrain in responders Less GM in the orbitofrontal cortex in non-responders; correlation of treatment response with the amount of orbito frontal GM
Li et al. (2025)	MOH (*n* = 33, 17 responders, 16 non-responders, before and 6 months after withdrawal) NC (*n* = 24)	Rs-fMRI	Simple analgesics (*n* = 6) Compound analgesics (*n* = 27)	MOH vs. NC Responders vs. non-responders	Increased FC in the left middle temporal gyrus of the left frontoparietal network Decreased FC in the left orbital inferior frontal gyrus of the left frontoparietal network
Sun et al. (2025)	MOH (*n* = 25) CM (*n* = 19) NC (*n* = 19)	sMRI DTI Rs-fMRI	Simple analgesics (*n* = 3) Triptan (*n* = 1) Combination analgesic (*n* = 21)	MOH vs. CM	Reduced GM in the parahippocampal gyrus and middle occipital gyrus, lower FA in the left cingulum bundle, decreased fALFF in the right putamen Negative correlation between fALFF in the right putamen with the frequency of acute pain medication use Enhanced FC between the right putamen and regions such as the frontal lobe, middle cingulate gyrus, lingual gyrus, and precuneus
Yuan et al. (2022)	MOH (*n* = 24) CM (*n* = 22) EM (*n* = 23) NC (*n* = 25)	Rs-fMRI	NR	MOH vs. CM	No significant differences
Pei et al. (2025)	MOH (*n* = 36) CM (*n* = 33) NC (*n* = 48)	QSM	NSAIDs (30.3%) Paracetamol (18.2%) Combination analgesics (15.2%) Triptans (12.1%) Traditional Chinese medicine treatments (12.1%) Combination of these medications (>30%)	CM vs. NC MOH vs. CM	Increased iron deposition in the caudate and putamen Greater iron deposition in the caudate, putamen, and globus pallidus
Meyer et al. (2017)	MOH (*n* = 14) NC (*n* = 15)	sMRI: VBM, Hippocampal tractography	NR	MOH vs. NC	Decreased volumes of left hemisphere temporal gyri and occipital middle gyrus, increased volume of the left inferior lateral ventricle Decreased number of fibers passing through the left hippocampus
Chen Z. et al. (2018)	MOH (*n* = 31) NC (*n* = 31	sMRI: VBM	NR	MOH vs. NC	Lower Hippocampal and Hippocampal subfields volume
Mahmut et al. (2023)	MOH (*n* = 10) EM (*n* = 10) CM (*n* = 10) NC (*n* = 30)	sMRI: VBM	NR	Patients vs. NC MOH vs. CM	Lower total hippocampal volume No significant difference
Dai et al. (2021)	MOH (*n* = 17) EM (*n* = 18) NC (*n* = 30)	Rs-fMRI: ICA	Combination analgesics	MOH vs. EM	Increased FC between bilateral habenula and salience network
Mehnert et al. (2018)	MOH (*n* = 18, before and 8 weeks after withdrawal) NC (*n* = 18)	sMRI: VBM rs-fMRI Task-based fMRI: nociceptive stimulation	Triptans (*n* = 5) NSAIDs (*n* = 12) Triptans + NSAIDs (*n* = 1)	MOH vs. NC Before vs. after withdrawal	Less volume in hippocampus, precuneus, inf. Frontal gyrus, bilateral OFC, medial orbital gyrus Stronger activation in left spinal trigeminal nucleus, right operculum and left posterior insula after withdrawal
Ferraro et al. (2012b)	MOH (*n* = 8, before and 6 months after withdrawal) CM (*n* = 8) HC (*n* = 8)	Task-based fMRI: decision-making paradigm	NSAIDs (*n* = 3) NSAIDs + Triptans (*n* = 4) Triptans + Caffeine (*n* = 1)	MOH vs. NC MOH vs. CM Before and after withdrawal	Reduced task-related activity in the substantia nigra/ventral tegmental area and increased activity in the ventromedial prefrontal cortex Reduced activity in the substantia nigra/ventral tegmental area Increased activity in the ventromedial prefrontal cortex
Beckmann et al. (2018)	MOH (*n* = 27, before and 6 months after withdrawal) HC (*n* = 27)	sMRI DTI	Simple analgesics (*n* = 26) Combined analgesics (*n* = 1)	MOH vs. NC Before and after withdrawal	No significant differences No significant differences

NC, normal control; MOH, medication overuse headache; EM, episodic migraine; CM, chronic migraine; sMRI, structural magnetic resonance imaging; rs-fMRI, rest state functional magnetic resonance imaging; DTI, diffusion tensor imaging; MRS, magnetic resonance spectroscopy; VBM, voxel-based morphometry; CBF, cerebral blood flow; CBV, cerebral blood volume; ICA, Independent component analysis; GMV, gray matter volume; FC, functional connectivity; NR, Not reported; NSAIDs, Non-Steroidal Anti-Inflammatory Drugs.

## 2 Pain matrix

The concept of “pain matrix” was introduced by Melzack (1999) suggesting that pain generation results from coordinated interaction among various brain regions rather than a single brain area. The traditional “pain matrix” comprises two separate but converging supraspinal pathways: the lateral and medial pain systems (Yao et al., 2023). The lateral pathway, consists of the thalamus, primary somatosensory cortex, secondary somatosensory cortex, and insular cortex (IC). The medial pathway includes the parabrachial nucleus, prefrontal cortex (PFC), anterior cingulate cortex (ACC), and amygdala (AMG). Additionally, the periaqueductal grey (PAG) and rostral ventromedial medulla (RVM) are key nuclei in the descending pain modulation pathway, transmitting modulatory information to the dorsal horn and trigeminal nucleus. Chronic pain perception in pathological states is considered to result from an imbalance in these pain modulatory pathways (Chen and Heinricher, 2019).

### 2.1 Lateral pain pathway

In a study utilizing voxel-based morphometry (VBM) analysis, researchers observed that patients with MOH exhibited a significantly greater gray matter volume (GMV) in the bilateral thalamus compared to NC, while decreased GMV in frontal regions including orbitofrontal cortex (OFC), ACC, bilateral IC, and precuneus (Riederer et al., 2012). A subsequent investigation by Yu et al. specifically examined thalamic subnuclei morphology in MOH patients versus NC, revealing increased GMV across all thalamic subnuclei (Chen et al., 2017c). Collectively, these two VBM studies consistently identify structural alterations in MOH patients, characterized by increased GMV in the bilateral thalamus, which might reflect central sensitization in chronic pain states.

Functional activity alterations are also observed in the lateral ascending pain pathway. One study performing fMRI during painful mechanical stimulation revealed reduced pain-related activity across the primary somatosensory cortex, inferior parietal lobule, and supramarginal gyrus (Ferraro et al., 2012a). Another study collecting fMRI in chronic migraine patients with MOH also demonstrated hypoactivity in the right supramarginal gyrus, right inferior and superior parietal cortex (Grazzi et al., 2010). The results of these two fMRI studies were both based on comparisons with NC. The study conducted by Chen et al. recruited NC, patients with episodic migraine (EM) and patients with MOH (Chen et al., 2017a). Patients with MOH showed decreased functional connectivity (FC) density in right parahippocampal gyrus, and increased FC density in left inferior parietal gyrus and right supramarginal gyrus compared to NC, while decreased FC density in right caudate and left insula compared to EM. The RSFC analysis further revealed that decreased FC in frontal-temporal–parietal pattern in MOH compared with EM. The changes in the right caudate and insula may represent characteristic alterations of MOH, rather than migraine itself. These fMRI studies indicate that MOH patients exhibit distinct functional alterations in the lateral ascending pain pathway, including hypoactivity in parietal and somatosensory cortical regions relative to NC, and specific FC reductions in the right caudate, left insula, and frontotemporal-parietal network compared to EM.

Fumal et al. (2006) measured glucose metabolism with 18FDG-PET in 68 NC and 16 CM with MOH patients. Their findings revealed hypometabolism in the bilateral thalamus, anterior cingulate gyrus, insula, and right inferior parietal lobule, while hypermetabolism was observed in cerebellar vermis. The functional and metabolic modification in the lateral pain system may be attributed to top-down regulation reducing painful inputs to the cortex, or to activity-dependent plasticity following excessive excitatory input induced by migraine attacks. The decreased thalamic metabolism identified in this study appears to contradict the increased thalamic volume mentioned earlier. Structurally, the increased GMV is attributed to compensatory gliosis (e.g., astrocyte proliferation) and neuronal remodeling in response to prolonged nociceptive input, enhancing the thalamus’s capacity to process sensory signals as a long-term adaptive change. Metabolically, reduced glucose uptake results from active top-down regulation to inhibit excessive thalamic neuronal activation and may also be influenced by medication-induced impairments. Thus, this phenomenon may represent a dual adaptive strategy: structural proliferation to cope with chronic stress and metabolic suppression to regulate overexcitation, reflecting the complexity of neural plasticity in MOH pathophysiology.

Using Magnetic Resonance Spectroscopy (MRS), Wang et al. analyzed metabolites along the thalamocortical pathway (Niddam et al., 2020). Their research demonstrated that MOH patients exhibited significantly elevated levels of glutamate and glutamine in the right mid-cingulate cortex, as well as increased myo-inositol in the left anterior cingulate cortex, compared to patients without MOH. Additionally, they identified a negative correlation between the myo-inositol laterality index in the anterior cingulate cortices and monthly acute medication use frequency. However, another MRS study focusing on brainstem indicated that medicine overuse showed no association with MRS ratios in CM patients (Lai et al., 2012).

### 2.2 Medial pain pathway

Using surface-based morphometry (Riederer et al., 2017), Sun and colleagues examined cortical thickness and gyrification in 29 patients with MOH and NC. Their analysis revealed reduced cortical thickness in the left prefrontal cortex and increased local gyrification in fusiform cortex, medial temporal regions, and the right occipital pole. Furthermore, enhanced gyrification in the right occipital pole indicated poor response following detoxification. Patients with MOH demonstrated relatively high arrival-time-corrected cerebral blood flow (CBF) and cerebral blood volume (CBV) values in the bilateral amygdala compared to CM, although the difference did not achieve statistical significance (Bai et al., 2023).

### 2.3 Descending pain pathway

In addition to ascending pain pathways, research has documented significant structural and functional alterations in descending pain pathways, with neuroimaging studies revealing morphological changes in key modulatory nuclei and disrupted FC patterns within corticospinal circuits. A previous study conducted by Riederer et al. (2012) found a significant increase of GMV in PAG in MOH patients compared with NC. This finding was corroborated by an independent investigation employing automated PAG volume measurement (Chen et al., 2017b). Notably, receiver operating characteristic (ROC) curve analysis revealed an optimal diagnostic cut-point of 0.341 mL for PAG volume, demonstrating 95.5% sensitivity and 63.6% specificity in differentiating MOH patients from NC. Further VBM analysis of the brainstem revealed that the primary site of PAG involvement was located in the ventrolateral subregion (Chen Z. Y. et al., 2018). Moreover, this study identified a significant positive correlation between left RVM volume and headache intensity as measured by the visual analog scale (VAS). However, the association between the altered GMV and emotion remains controversial. One study reported a positive association between GMV in PAG and anxiety, whereas two other studies found no significant correlation between psychological scale scores and brainstem GMV. These findings demonstrate prominent structural alterations in key nuclei of the descending pain pathway in MOH patients, including increased PAG volume in MOH, which may result from neuroadaptive responses to chronic pain and medication overuse. This is supported by findings that PAG volume normalizes following successful drug withdrawal, suggesting a reversible, compensatory change within the brain’s pain control system (Riederer et al., 2013).

### 2.4 Other pain associated regions

Some studies have focused on brain regions that, while not part of the traditional pain matrix, are involved in generating pain signals. The marginal division of neostriatum (MrD), discovered in 1988, has been found involved in pain modulation due to exclusively localized nociceptive neurons (Chudler et al., 1993; Chudler and Dong, 1995). Decreased FC of MrD was detected from EM to CM, from EM to MOH, and from CM to MOH, and increased FC was observed from EM to CM and from CM to MOH. The results demonstrated that MrD played a key role in migraine chronicization (Chen et al., 2016).

The hypothalamus is closely connected to other brain regions belonging to the pain matrix such as the amygdala, the periaqueductal gray and the rostral ventromedial medulla (Xie and Dorsky, 2017). Using state-of-the-art 3D pseudo-continuous arterial spin labeling (PCASL) MR imaging, hypothalamic subunits presented with significantly decreased perfusion in the left posterior, left tubular superior, right anterior–inferior, right tubular inferior, right tubular superior, right posterior subunit and the entire right hypothalamus (Liu M. et al., 2024).

Compared to NC, FC with the left precuneus of MOH patients was decreased in pain processing regions (right frontal cortex, angular gyrus, left post central and supramarginal gyri), but increased in memory processing areas (right hippocampus, left superior frontal cortex and left fusiform gyrus). When compared to EM, MOH patients displayed decreased FC within the DMN, together with increased connectivity between the left precuneus and regions involved in memory processing. The result demonstrated that the decreased connectivity within the DMN and the increased connectivity with a memory processing network at rest seem to be more specific to MOH. However, these functional modifications were not accompanied by significant gross morphological changes (Chanraud et al., 2014).

## 3 Mesocorticolimbic dopaminergic circuit

Beyond alterations in pain-processing networks, MOH also involves significant dysregulation of brain reward circuitry. Previous studies indicate that MO shares specific behavioral, genetic, and neuronal pathways with drug addiction (Takahashi et al., 2021). The mesocorticolimbic dopaminergic circuit (also known as the reward system) comprising ventral tegmental area (VTA), nucleus accumbens (NAc), dorsal striatum (putamen and caudate nucleus), PFC (especially OFC), AMY and hippocampus, represents the primary neurological substrate of drug addiction (Haber and Knutson, 2010). Research demonstrates that the mesolimbic dopaminergic circuitry participates acute and chronic pain modulation (Waung et al., 2019; Kuner and Kuner, 2021).

VBM analysis revealed decreased GMV in VTA and bilateral substantia nigra among MOH patients (Chen Z. Y. et al., 2018). Similar to individuals with substance use disorders who exhibit steeper discounting behavior, MOH patients demonstrate comparable characteristics and diminished task-related responses (Niddam et al., 2023). Task-related imaging revealed reduced encoding of subjective value in the dorsal medial PFC during temporal discounting and in the ventral striatum and ventral medial PFC during impulsive decision making. Additionally, decreased FC between ventral medial PFC and the bilateral ventral striatum correlates with acute analgesic use frequency. Furthermore, classification algorithms effectively distinguish MOH from other headache forms based on FC spatial patterns in dorsal and ventral striatal regions of interest (Torta et al., 2016).

The OFC, prefrontal cortex component that regulates control behavior based on potential consequences, shows alterations in drug addicts (Schoenbaum et al., 2006; Schoenbaum and Shaham, 2008). Studies demonstrate disrupted white matter integrity and reduced GMV in orbitofrontal cortex among MOH patients compared to CM without MOH, correlating with analgesics use frequency (Lai et al., 2016; Tantik Pak et al., 2021). Through dopamine transporter PET, Liu H. et al. (2024) identified potential compensatory downregulation in medial OFC rather than lateral OFC (). Follow-up data indicated that patients exhibiting more pronounced orbitofrontal cortex decline demonstrate poorer treatment respondence (Riederer et al., 2013). Functional analysis revealed that decreased FC in the left orbital inferior frontal gyrus of frontoparietal network correlates with MOH treatment prognosis (Li et al., 2025). These studies demonstrated that the OFC of MOH patients presents disrupted white matter integrity, reduced GMV, dopamine transporter downregulation as well as decreased FC, which are closely associated with analgesic use frequency and prognosis—highlighting the OFC as a key brain region linked to MOH pathophysiology and clinical outcomes.

A recent multimodal MRI study examined differences in GMV, white matter integrity and functional activity between CM and MOH patients. The CM with MOH group showed significantly reduced GMV in parahippocampal and occipital regions and fractional anisotropy in the cingulum bundle. The study demonstrated decreased fALFF in the right putamen and enhanced FC with regions including the frontal lobe, middle cingulate gyrus, lingual gyrus, and precuneus, potentially representing a compensatory response. It is noteworthy that these functional activity and connectivity alterations negatively correlate with acute analgesic use frequency (Sun et al., 2025). Existing studies have reported inconsistent findings regarding caudate nucleus FC changes in MOH patients. One study using bilateral caudate nuclides as seed found no significant differences between CM patients with and without MOH (Yuan et al., 2022). In contrast, another study demonstrated decreased FCD in right caudate in MOH compared to EM (Chen et al., 2017a). This discrepancy may be attributed to different control groups employed in the two studies. Additionally, Yuan et al. observed that CM patients had higher FC values between the bilateral precuneus, left anterior cingulate gyrus, right middle cingulate cortex, right lingual gyrus, and right caudate nucleus compared to EM patients, suggesting caudate functional alterations may originate from headache progression, rather than medication overuse. Quantitative susceptibility mapping (QSM) analysis revealed increased iron in MOH patients compared to CM, primarily in caudate, putamen, and globus pallidus. Notably, this deposition correlates with monthly medication frequency rather than headache burden (Pei et al., 2025). As iron facilitates dopamine synthesis, these findings further support the correlation between MOH and drug addiction (Hare and Double, 2016; Juhas et al., 2017).

Hippocampus, as a part of limbic system, serves a crucial role in pain modulation, activated during pain processing and nociceptive stimuli modification (Mutso et al., 2012). MOH patients show significantly lower hippocampus and subfields volume compared to NC, except in right hippocampus tail, bilateral parasubiculum, and hippocampus fissure (Chen Z. et al., 2018). Hippocampal seed-based tractography revealed 29% fewer reconstructed fibers from the left hippocampus in MOH compared to NC (Meyer et al., 2017). However, a comprehensive study including NC, EM, CM, and MOH patients found that while total hippocampal volume decreased in patient groups versus controls, no significant differences emerged among patient subgroups. Though lower volumetric values were found in MOH compared to the NC, there were no statistically significant differences (Mahmut et al., 2023). Previous studies on hippocampal volume in EM and CM patients have yielded inconsistent results. Specifically, some studies indicate adaptive plasticity at lower headache frequencies, while higher frequencies lead to maladaptive reduction in the hippocampal volume (Maleki et al., 2012; Liu et al., 2017; Yu et al., 2021). This volume fluctuation according to headache attack frequency may be one of the factors contributing to the discrepancies in hippocampal volume findings across different studies. Additionally, in Mahmut et al.’s study, each subgroup included only 10 participants—insufficient sample size that may also result in false negative outcomes.

The habenula, an anti-reward system core component, receives input from limbic-forebrain and projects to midbrain nucleus. Its unique anatomical position enables the habenula to serve as a hub integrating value-based, sensory, and experience-dependent information to regulate various motivational, cognitive, and motor processes (Matsumoto and Hikosaka, 2007; Hu et al., 2020). Recent studies have established a role for the habenula in drug addiction, including addiction to morphine, cocaine, and other substances (King et al., 2022; Fu et al., 2023). Independent component analysis (ICA) analysis revealed elevated FC between bilateral habenula and the salience network in patients with MOH compared to EM and NC. Correlation analysis demonstrated significant association between medication overuse duration and habenula-salience network connectivity (Dai et al., 2021). The findings suggest that dysfunction within the habenula-substantia nigra (SN) circuit is likely to compromise cognitive control over medication intake, and the length of time spent in a state of medication overuse is associated with enhanced synchronous neuronal activity.

## 4 Alterations after withdraw

Withdrawal of the offending drug (s) is considered the initial step in MOH management. Several studies have explored alterations in brain structure and function in MOH patients before and after drug withdrawal. Research detected hypoactivity in the right supramarginal gyrus, right inferior and superior parietal cortex involving the lateral pain pathway in MOH patients compared to NC when exposed to pressure stimulation at specific intensity levels via fMRI. The hypoactivity returned to nearly normal level 6 months after withdrawal of the offending medications (Grazzi et al., 2010), aligning with findings from the study conducted in 2012 (Ferraro et al., 2012a). A longitudinal sMRI study provided insights regarding which types of patients with MOH respond effectively to medication withdrawal (Riederer et al., 2013). Results indicated that only patients showing significant clinical improvement demonstrated a notable decrease in previously increased gray matter in the midbrain including PAG and nucleus cuneiformis, while non-improving patients showed no changes. Additionally, non-responders exhibited less GMV in the OFC at baseline, with GMV in this region positively correlating with treatment response. Notably, GM changes in reward circuitry structures, such as the ventral striatum persisted after detoxification. These findings suggest that MOH patients responding to detoxification treatment display adaptive GM changes within pain modulatory systems, potentially reflecting neural plasticity.

Several studies focused on the mesolimbic reward circuitry, particularly the OFC. Using a well-established trigeminal nociceptive fMRI paradigm, the OFC regained FC to the nociceptive input region and the cerebellum after withdrawal (Mehnert et al., 2018). However, these studies did not include patients with CM. Ferraro et al. (2012b) conducted a longitudinal follow-up study that included CM patients, observing reduced task-related activity in the substantia nigra/ventral tegmental area complex and increased activity in the ventromedial prefrontal cortex compared to NC and reduced activity in the substantia nigra/ventral tegmental area complex compared to CM. While ventromedial PFC dysfunctions partially recovered after withdrawal, substantia nigra/ventral tegmental area complex dysfunctions persisted, potentially indicating vulnerability to medication overuse. The FDG-PET study revealed similar results, with most dysmetabolic areas recovering to almost normal glucose uptake post-withdrawal, except the OFC, which showed further metabolic decrease. Sub-analysis indicated that orbitofrontal hypometabolism primally result from overuse of combination analgesics and/or ergotamine-caffeine preparations. These findings suggest that headache-induced structural and functional changes may be reversible, while alterations from excessive drug addiction are persistent, potentially explaining high relapse rates in MOH patient (Fumal et al., 2006).

However, one study using sMRI and DTI analysis found no significant changes in gray and white matter structures before and after medication withdrawal, nor differences between MOH and NC. This possible discrepancy reason might relate to the predominant use of single analgesics among included MOH patients. Future research should further investigate the impact of different medication types (Beckmann et al., 2018).

In summary, drug withdrawal leads to the reversal of some pain-processing pathway-related structural and functional brain abnormalities in MOH patients, but reward-associated alterations (e.g., in the SN/VTA complex and OFC) often persist.

## 5 Discussion

Current research demonstrates structural and functional alterations in pain-processing pathways and the mesolimbic circuitry in MOH patients compared with migraineurs and NC, showing partial reversibility after drug withdrawal. The specific finding includes: (1) Within the pain matrix, increased thalamic volume and descending pathway structures such as the PAG in the midbrain occur, while GMV decreases in the insula and primary sensory cortex, with generally reduced FC, possibly indicating a compensatory downregulation; (2) Key mesolimbic system regions primarily exhibit reduced GMV and decreased functional activity; (3) After drug withdrawal, pain processing related alterations may recover, while changes in addiction-associated brain regions associated, such as the orbitofrontal gyrus, persist.

The present review is premised on the classical conception of the “pain matrix, “defined by the consistent activation of a core set of regions (S1/S2, ACC, insula, thalamus, and PFC) in early neuroimaging investigations. However, the specificity of this network has been contested, as similar activations are observed during various non-nociceptive tasks, suggesting it may represent a broader salience-detection system. This has prompted a conceptual evolution, with recent research moving beyond the idea of fixed clusters to focus on the dynamic connectivity and interplay between large-scale, multi-modal networks (Legrain et al., 2011; Cui et al., 2015). Studies of patients with MOH have revealed a complex pattern of neural changes, including increased thalamic volume co-occurring with reduced metabolism and function in other regions of the traditional pain matrix, such as the primary/secondary somatosensory cortices and the insula. These findings are suggestive of central sensitization and compensatory mechanisms. Concurrently, alterations observed in the default mode and memory-related networks when MOH patients are compared to EM cohorts indicate that the chronicization process involves multimodal networks rather than being confined to the traditional pain matrix. However, as the cited studies often lack comparison with CM, it remains unclear whether these network changes are specifically tied to chronicization or are a direct consequence of excessive medication.

Similarly, the mesocorticolimbic dopaminergic circuit, central to motivation and reward, plays a complex and dynamic role in pain modulation and addiction. Increasing evidence shows pain-induced reorganization of the reward circuitry, which contributes to the transition from acute to chronic pain (Navratilova and Porreca, 2014). Meanwhile, dysregulation of dopamine function in the brain reward center may further promote comorbid mood disorders and vulnerability to addiction (Serafini et al., 2020). Patients with MOH exhibit reduced volume of the ventral striatum and decreased metabolic function of the OFC, and these changes are difficult to reverse after drug withdrawal—suggesting persistent dysfunction in the reward system. These maladaptive changes are consistent with studies showing that both addictive drugs and aversive stimuli can trigger plasticity at inhibitory synapses within the reward circuit. Iron, an essential element for dopamine synthesis, has been found to accumulate in the caudate and putamen of MOH patients as revealed by QSM. This finding further supports the association between MOH and addiction-related neuropathology. The accumulation may be attributed to the neurotoxic effects of chronic medication overuse, which could compromise blood–brain barrier integrity and facilitate iron deposition (Yamamoto and Raudensky, 2008). Furthermore, as evidenced by fMRI studies, such iron overload may subsequently alter functional activity and connectivity within these regions, contributing to the dysfunction of reward circuits in MOH.

The origin of structural and functional alterations in the pain matrix and reward system of patients with MOH—whether they result from medication overuse or from the chronicization of headache—and the direction of causality between these neural changes and medication overuse remain to be fully elucidated. Existing comparative studies between patients with MOH and those with migraine (particularly chronic migraine, CM), as well as longitudinal follow-up studies, may help clarify these issues. Specifically, when compared to CM patients, those with MOH show more pronounced changes in reward-related regions such as the ventral striatum, OFC, and putamen, including disrupted functional connectivity, decreased neural activity, gray matter volume reduction, and impaired white matter integrity. Although these alterations have been tentatively linked to medication overuse, some studies report no significant differences between MOH and CM, underscoring the need for further validation. Longitudinal assessments before and after drug withdrawal further reveal that structural and functional alterations in areas such as the OFC and the SN/VTA persist even after discontinuation of medication. Notably, reduced OFC volume is correlated with poor treatment response in MOH, suggesting that it may be both a consequence of overuse and a factor promoting further use, supporting a bidirectional causal model. However, these findings should be interpreted with caution due to the generally limited sample sizes in existing follow-up studies. Recent preclinical evidence from a voluntary rizatriptan intake model of MOH reinforces the involvement of specific brain networks (Gong et al., 2023). Network-level analysis identified the prelimbic cortex (PrL) and the spinal trigeminal nucleus caudalis (SPVC) as central hubs. The PrL demonstrated broad functional connectivity with regions implicated in addiction, such as the insular cortex and nucleus accumbens, whereas the SPVC was predominantly linked to pain-processing areas. The decrease in c-Fos activation in these regions following withdrawal supports the conclusion that these functional alterations result from medication overuse. Despite these insights, the core questions regarding etiology and causality in MOH remain unresolved. Future large-sample longitudinal studies that include both EM and CM cohorts, combined with translational experimental approaches, will be essential to fully disentangle these complex mechanisms.

Equally critical is the question of whether distinct analgesic classes drive distinct neuropathological pathways. A direct comparison of different medication classes is currently lacking in MOH research, as most studies feature heterogeneous patient cohorts. Nevertheless, several studies focusing predominantly on simple or combination analgesic overuse deserve consideration. One study of simple analgesic overusers reported decreased FC in the right caudate and left insula in MOH compared to EM. In contrast, a separate DTI study, also investigating simple analgesic overusers, did not detect significant differences between MOH patients and NC. Among cohorts dominated by combination analgesic overuse, alterations in MOH patients are predominantly observed within the reward system. These include changes in dopamine transporter availability in the orbitofrontal cortex, functional connectivity of the habenula and salience network, and functional activity in striatal regions such as the putamen. Currently, there is a notable lack of studies specifically focusing on triptan-overuse MOH patients. Addressing this gap requires future research that conducts direct, systematic comparisons of patients overusing different analgesic classes to elucidate their distinct brain mechanisms.

Various studies have found alterations in “pain matrix” and reward system in MOH and may provide potential biomarkers and therapeutic targets for clinical management. For example, the volume and metabolism of the OFC fail to recover even after drug withdrawal and the observed reduction in its volume specifically suggests a poor response to withdrawal therapy. This suggests that future personalized treatment strategies could be guided by the structural, functional, and metabolic characteristics of the OFC, including non-invasive neuromodulation approaches such as TMS targeted at this region for refractory MOH. Meanwhile, there is currently a lack of neuroimaging studies focusing on MOH relapse. Future longitudinal research should aim to develop relapse prediction models, enabling better personalized and stratified management of MOH patients. Collectively, current research lays a foundation for developing prognostic and relapse prediction models in MOH, as well as for designing personalized management strategies. However, further advances critically depend on larger-scale, longitudinal studies that incorporate well-defined subgroups—including EM, CM, and MOH patients stratified by medication overuse type.
